# Mechanical Properties and Microstructural Characterization of Concrete with Recycled Aggregates from 20-Year Marine-Exposed Structures

**DOI:** 10.3390/ma19173714

**Published:** 2026-08-31

**Authors:** Gustavo Adolfo Mendoza-Martínez, Fausto A. Canales, Heidis Cano

**Affiliations:** 1 Department of Civil and Environmental, Universidad de la Costa, Barranquilla 080002, Colombia; gmendoza1@cuc.edu.co; 2 Civil Engineering Program, Faculty of Engineering, Universidad Militar Nueva Granada, Campus Cajica, Cajica 250247, Colombia

**Keywords:** construction and demolition waste, compressive strength development, microstructural characterization, circular economy in concrete

## Abstract

**Highlights:**

- RCA derived from a 20-year marine-exposed pier showed limited petrographic evidence of deterioration.
- Concrete with 50% RCA and a low W/C ratio achieved compressive strength above design values.
- The source concrete microstructure was associated with the strength development of RCA-based mixes.

**Abstract:**

This study evaluated the mechanical properties and microstructural characteristics of concrete made with recycled concrete aggregates (RCAs) derived from a pier exposed to a tropical marine environment for more than 20 years. The RCA was characterized physically, chemically, and mechanically to assess porosity, inherited chemical contamination, and potential degradation mechanisms associated with prolonged marine exposure. New concretes were produced with 50% replacement of natural coarse aggregates by RCA and water–cement (W/C) ratios of 0.67, 0.61, and 0.47. Compressive strength was measured at 3, 7, and 28 days. The mixture with 50% RCA and W/C = 0.47 achieved a 28-day compressive strength of ≈22.8 MPa, exceeding the design strength of 21 MPa. The source concrete and derived RCA exhibited a thin-section air-void content of 3.3%, acid-soluble chloride content of 0.074% by mass of concrete, and SO_3_ content of 1.15%. Petrographic examination identified no evidence of harmful alkali–silica reactivity in the examined material. Statistical analysis of the experimental results indicated increasing compressive strength with decreasing W/C ratio and increasing curing age. These findings demonstrate that, under the investigated mixture proportions and curing conditions, 50% RCA replacement can achieve the specified compressive strength. They also indicate an association between the source concrete’s inherited microstructural characteristics and the strength development of the recycled aggregate mixes.

## 1. Introduction

The construction sector is among the industries with the greatest environmental impact globally, accounting for around 25% of worldwide solid waste production and consuming 40% of all extracted natural resources [1,2,3]. Additionally, between 30% and 40% of all solid waste is deposited in landfills worldwide [4]. In this context, the search for sustainable solutions has become increasingly important, especially with the adoption of circular economy (CE) principles to reduce reliance on virgin materials and encourage the reuse of Construction and Demolition Waste (CDW). The CE has become a key concept for promoting sustainability, driving more responsible urban development, and improve environmental protection [5], and for reconciling the pressures of production systems on the environment and the consumption of natural resources [6,7].

The reuse of concrete is one of the most promising strategies within this approach, as it reduces the extraction of natural aggregates, decreases waste generation, and mitigates the environmental impact associated with infrastructure demolition [8,9]. Current trends in CDW reuse indicate rates ranging from 25% to 70% [10,11,12]. However, the use of recycled concrete aggregates (RCAs) presents technical challenges due to significant variability in quality [13,14], lack of quality control [15], absence of regulations [16,17], the time required for on-site protection [18], the lack of large, reliable material supplies [17], high water absorption, increased porosity, lower strength, and weak interfacial transition zones caused by adhered mortar [19,20]. Beyond the technical aspects, RCA adoption is further constrained by environmental and economic barriers such as limited commercialization and policy gaps [17,21], higher processing costs and logistical constraints [17,22], and uncertain market acceptance among construction stakeholders [19], which together hinder large-scale implementation despite its sustainability potential.

These aforementioned limitations are compounded by performance variability tied to processing and mix design [19], as well as the need to optimize mixture formulations due to low pH values and associated compressive strength issues [23]. These challenges are particularly critical for infrastructure exposed to aggressive environments, such as marine conditions, where the presence of chlorides and sulfates adversely affects concrete durability [24,25,26], thereby increasing corrosion risk in reinforced structures [27,28,29].

Studies have shown that the performance of concrete containing RCA, especially in aggressive environments such as marine settings, depends on the quality of the parent material and the mix design. RA properties are linked to the original concrete’s strength and condition; high-strength sources may retain performance, while lower-quality ones can reduce strength by up to 30% and increase porosity, allowing chloride ingress [30]. Belal et al. [31] investigated the performance of RCA in underwater concrete, finding that its inherent porosity and higher water absorption compared to natural aggregates can increase chloride permeability and reduce workability. Although this porosity is a key limitation, it can be mitigated through specific mix strategies. In this regard, several authors have shown that adding supplementary cementitious materials (SCMs) and reactive additives improves mechanical and durability performance. For example, slag, red mud, and glass powder can compensate for RCA deficiencies, allowing up to 50% replacement to improve strength and chloride resistance at higher SCM levels [32]. Metakaolin and nanosilica can refine the pore structure and increase chloride binding, thereby ensuring durability at early ages under aggressive conditions [25]. The use of magnetized water also enhances hydration and reduces microvoids, thereby improving compactness [33].

At the structural scale, experiments on beams, columns, and frame systems with varying RCA contents have shown that members with moderate RCA replacement can achieve cyclic, fatigue, and seismic performance comparable to conventional concrete, provided that mix design and detailing are adapted to the properties of the recycled aggregate [34,35].

Additionally, using RCA from chloride-contaminated sources does not necessarily compromise early microstructural properties or basic durability, but it may affect long-term chloride resistance, requiring design measures to prevent reinforcement corrosion [36]. These findings suggest that the early-age quality of concrete can be maintained even when materials containing contaminants are used, because the presence of chlorides or sulfates does not necessarily cause significant reductions in mechanical properties at 28 days. This behavior has been confirmed by Debieb et al. [37], who reported that different types of contamination had no substantial effect on compressive strength, tensile strength, or modulus of elasticity ($E_{c}$) at early ages. However, the same study also highlights that concrete produced with 100% RCA exhibits inherently lower mechanical performance than conventional concrete, with reductions of up to 40% in compressive strength, 19% in splitting tensile strength, and 38% in $E_{c}$ compared with concrete made with natural aggregates.

Despite these advances, scientific evidence remains limited on the use of RCA sourced from structures exposed to real marine environments over extended service periods, as most available studies rely on accelerated or simulated exposure conditions. Therefore, the aim and contribution of this study are to evaluate the mechanical properties of concrete produced with RCA derived from concrete structures that were exposed to a tropical marine environment for more than 20 years.

## 2. Materials and Methods

### 2.1. Study Area

**Figure 1:**
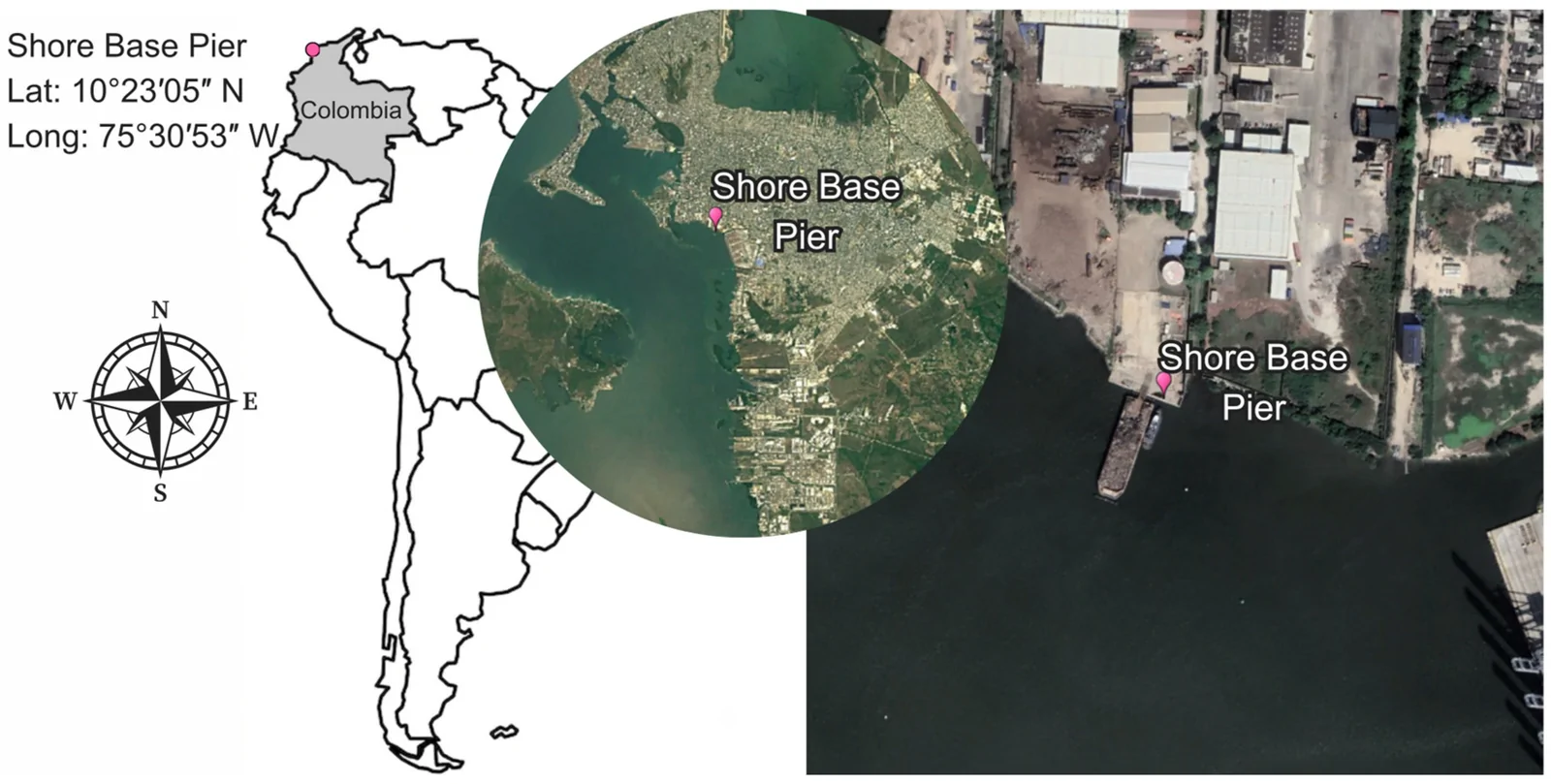
Location of the Shore Base Pier (SBP).

**Figure 2:**
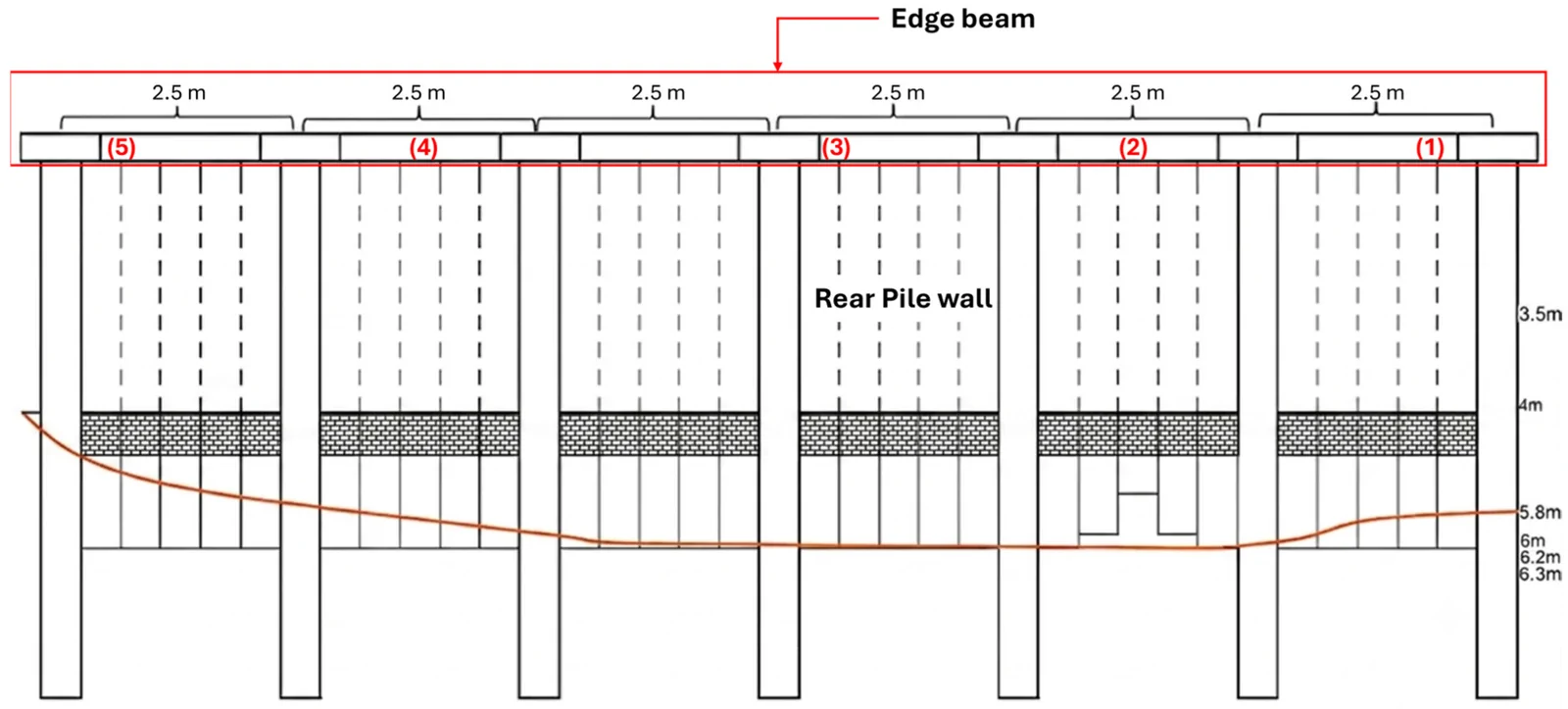
Longitudinal profile of the Shore Base Dock with numbers in red indicating the five locations along the edge beam where rebound hammer tests were conducted.

The samples used in this study were obtained from a reinforced concrete pier located in the port and industrial sector of Cartagena Bay, Colombia (Figure 1). The Shore Base Pier (SBP) was built in the late 1990s with a design compressive strength ($f_{c}^{\prime }$) of 21 MPa (3000 psi), and its demolition process began in 2024. The samples were taken in December 2023. The SBP was approximately 50 m long and was exposed to a tropical marine environment [38] throughout its service life. Figure 2 shows the longitudinal profile of the study area, including the pile embedment in the ground profile and the locations of the five points where rebound hammer tests [39] were conducted to determine in situ concrete strength. Subsequently, core extraction was conducted in the vicinity of these five points, yielding six representative core samples (with Zone 5 represented by two cores, Zone 5a and Zone 5b) for mechanical and durability testing. The geographic coordinates of the SBP are approximately 10°23′05″ N and 75°30′53″ W.

### 2.2. Characterization of the Study Material

**Figure 3:**
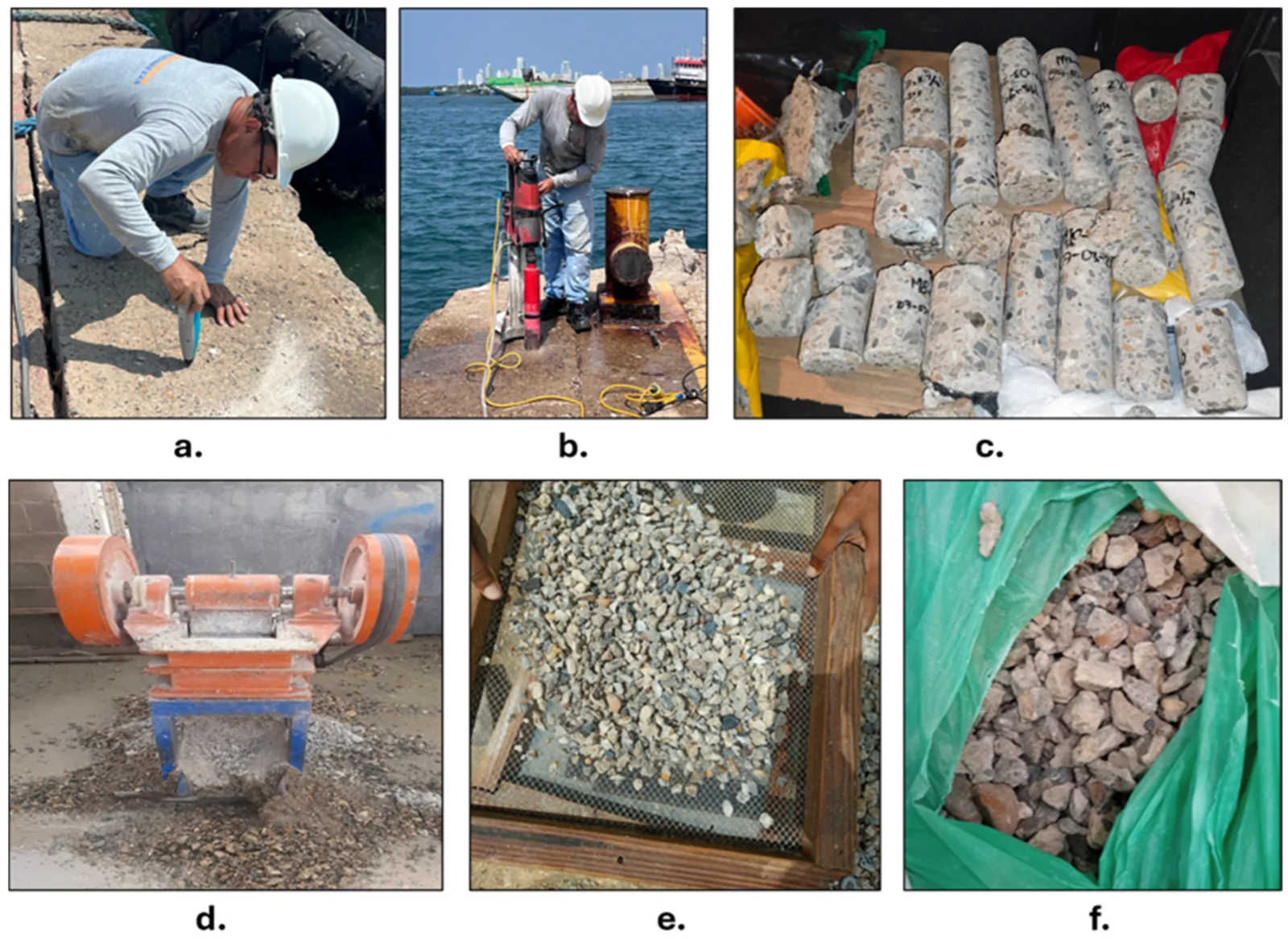
Process of obtaining recycled concrete aggregates (RCAs) after the crushing process. (**a**) sclerometric test, (**b**) core extraction process, (**c**) concrete cores obtained from SBP, (**d**) jaw crushing machine, (**e**) 3/8″ gravel, and (**f**) 1/2″ gravel.

The surface strength of the concrete was evaluated using the sclerometer test (Figure 3a) in accordance with ASTM C805 [39] across the 5 test zones in Figure 2. In addition, core samples were extracted from the pier structure (Figure 3b) for a total of 12 samples, following the guidelines of ASTM C42 [40]. These cores were classified into five evaluation zones (with Zone 5 represented by two sub-samples, Zone 5a and Zone 5b), each representing a distinct segment of the edge beam, to link mechanical performance to location along the structure. The length and diameter of each core were measured before testing, and the L/D ratio was calculated for each specimen. Compressive strength was calculated by dividing the maximum load by the cross-sectional area calculated from the individually measured diameter. For cores with L/D ≤ 1.75, the measured strength was multiplied by the applicable L/D correction factor in accordance with ASTM C42 [40]. No L/D correction was applied to cores with L/D > 1.75.

From these cores, porosity (permeable voids), absorption, and density were determined according to ASTM C642 [41]. Furthermore, durability-related parameters were evaluated by determining chloride [42] and sulfate [43,44,45] concentrations. The advancement of the carbonation front and the petrographic characteristics of the concrete were evaluated in accordance with ASTM C856 [46] to evaluate its physical and chemical condition in service, identify the primary components of the cement matrix and the aggregate composition, and assess the potential for alkali–silica reaction (ASR). This thin-section analysis was carried out using a Motic BA310Pol transmitted-light petrographic microscope (Motic, Xiamen, China) equipped with a Moticam 10.0 camera (Motic, Xiamen, China), with approximately 300 points counted.

In summary, the structural characterization of the pier followed a methodology that progressed from a preliminary surface evaluation to a more detailed laboratory analysis. Initially, the five specific locations shown in Figure 2 were established along the edge beam for non-destructive sclerometry tests to identify the surface resistance. To better represent the structure’s internal properties, the scope was subsequently focused on five study zones adjacent to the initial testing points. While a total of 12 concrete cores were extracted from the pier to ensure sufficient material volume, six representative core samples (one from each of Zones 1–4, and two from Zone 5, designated as Zone 5a and Zone 5b) were selected to provide the mechanical and physical data reported in this study.

**Figure 4:**
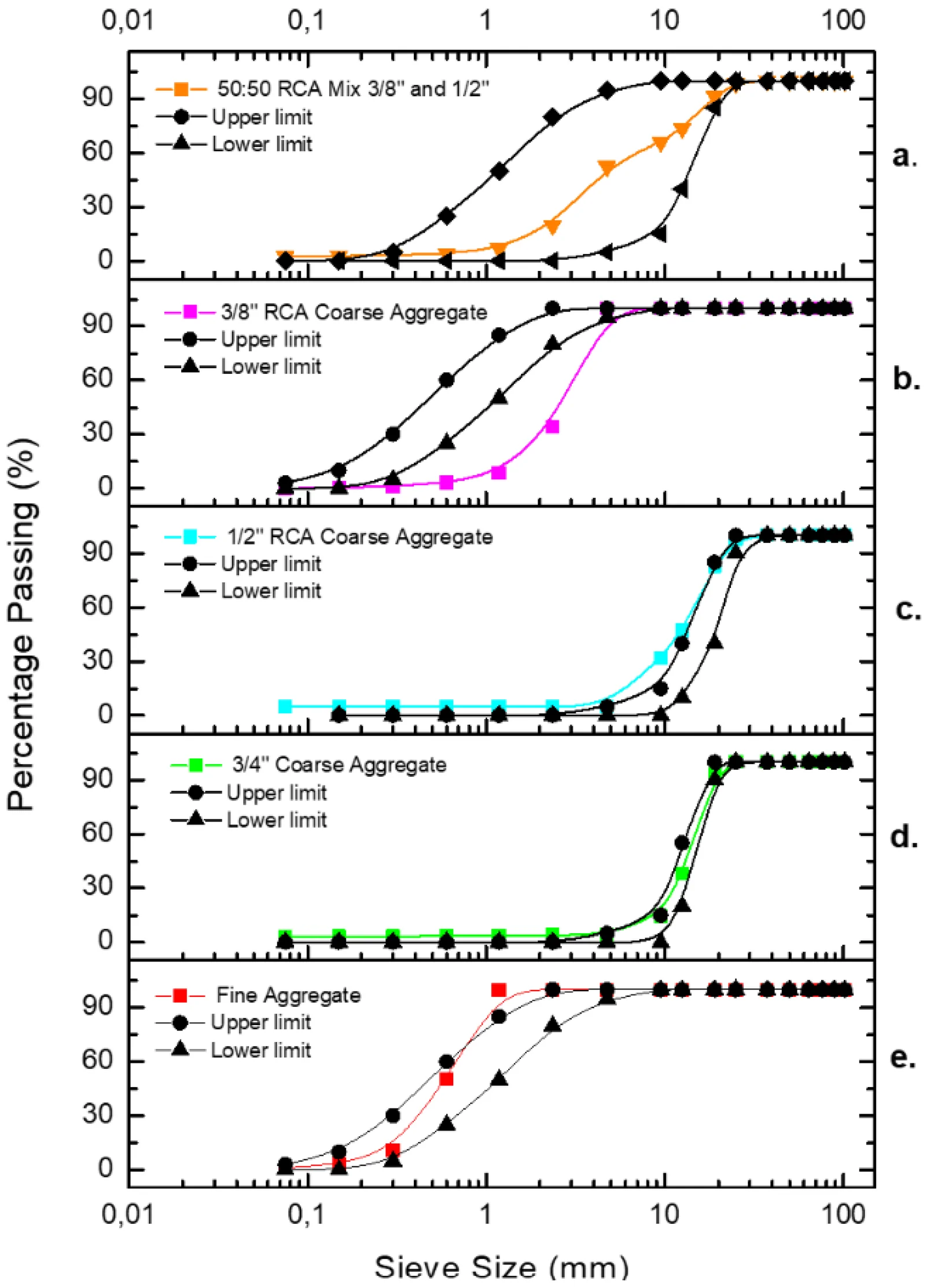
Particle size distribution graph of aggregates employed in the concrete fabrication. (**a**) 50:50 RCA mix 3/8″ and 1/2″, (**b**) 3/8″ RCA coarse aggregate, (**c**) 1/2″ RCA coarse aggregate, (**d**) 3/4″ coarse aggregate from the Arroyo de Piedra quarry (Luruaco, Colombia), (**e**) fine aggregate from the Rotinet quarry (Repelón, Colombia).

The concrete cores (Figure 3c) were crushed to obtain RCAs. Using a jaw crushing machine (Figure 3d), coarse aggregate fractions with nominal sizes of 3/8″ and 1/2″ by sieving (Figure 3e,f) were obtained, according to ASTM C33 [47]. Density, water absorption [48], resistance to degradation (Los Angeles Abrasion test) [49], and particle size distribution [50] were characterized for the aggregates employed in this study (see Figure 4). Additional details for the aggregates employed (from RCA and quarries) in the fabrication of new concrete with RCAs are presented in Table 1.

Because the initial particle size distribution of the 1/2″ coarse RCA did not meet the grading requirements of ASTM C33 [47], it was blended in equal parts (50–50%) with 3/8″ RCA. This adjustment ensured that the combined material met the particle size distribution limits for a nominal 3/4″ coarse aggregate, as required for the concrete mixtures in this study. The resulting gradation of this combined 3/8″ and 1/2″ blend is illustrated in Figure 4a. This standardized aggregate was subsequently used to determine bulk density and void content in accordance with ASTM C29 [51] for concrete mix proportioning.

### 2.3. Preparation of New Concretes with RCA

Concrete cylinders measuring 100 × 200 mm were manufactured with a $f_{c}^{\prime }$ = 21 MPa, in accordance with ASTM C192 [52]. Table 2 shows the mix design used to prepare the cylinders. Type I Portland cement (Argos, Medellín, Colombia), fine sand from the Rotinet quarry, and natural coarse aggregate from the Arroyo de Piedra quarry, all located in the department of Atlántico, Colombia, were used; the fine and coarse aggregates complied with the technical specifications of ASTM C33 [47]. Potable water was used for all mixtures. Three water–cement (W/C) ratios were adopted to study the influence of mix proportioning on mechanical performance and workability: 0.67 (M1), 0.61 (M2), and 0.47 (M3). These three mixtures were designed to subsequently analyze the relationship between compressive strength and the water–cement ratio.

### 2.4. Statistical Analysis

Compressive strength results for the cylinders were analyzed using Minitab v.19.1.1 [53] and JASP v.0.95.3 [54] to evaluate the effects of curing time and W/C ratio.

## 3. Results

### 3.1. Concrete Characterization

#### 3.1.1. Compressive Strength of Concrete

The compressive strength test results for the extracted cores are shown in Table 3. Values ranged from 11.8 MPa to 33.9 MPa, with an average of 23.6 MPa and a standard deviation of 8.8 MPa. Some cores were extracted from areas with repairs and structural reinforcements, where the design strength was unknown, and showed values lower than the $f_{c}^{\prime }$ of the edge beam (21 MPa). The average compressive strength of the six tested cores was 23.6 MPa, exceeding the nominal design strength of 21 MPa. However, the individual results showed substantial spatial variability, ranging from 11.8 to 33.9 MPa. Zone 3 had a corrected compressive strength of 11.8 MPa, below 75% of the nominal design strength; therefore, the core results do not support an unqualified assessment of structural adequacy for all investigated locations under Colombian standards [55].

**Figure 5:**
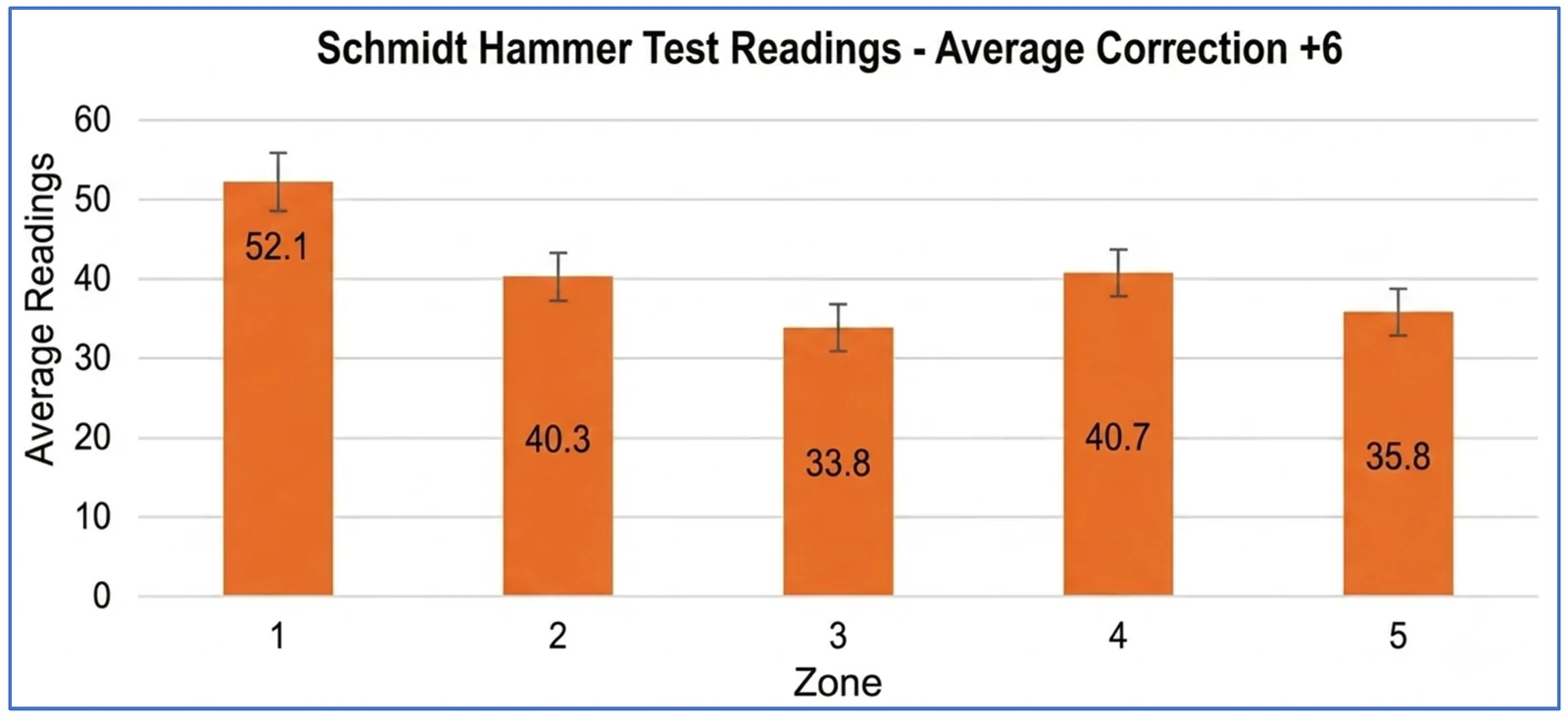
Average rebound values from the sclerometer tests in each sampling zone, corrected according to [39].

Figure 5 presents the sclerometer test results, showing the average rebound values obtained in each sampling zone. The measurements show noticeable variation, likely due to local surface conditions. In total, multiple rebound readings were taken at each of the five test zones along the edge beam and analyzed in accordance with ASTM C805 [39]. For each zone, an initial average rebound number was calculated, and individual readings that differed from this average by more than six units were discarded; if more than two readings in a set fell outside this ±6 unit range, the entire set was rejected and remeasured, in accordance with Section 9 of the standard [39]. After this filtering process, a corrected average rebound number was obtained for each zone, as shown in Figure 5, along with the corresponding measurement dispersion. These corrected averages were then used to estimate in situ compressive strength using the rebound-strength correlation adopted in this study, providing a consistent basis for comparing the surface condition of the concrete along the edge beam. The lowest values were observed at the sampling points in Zones 2, 3, and 5, corresponding to the edge beam in the intermediate spans of the south zone and the final span of the north zone, which are the concrete elements with the lowest structural stiffness.

#### 3.1.2. Physical, Chemical, and Petrographic Analysis of Concrete

For a thin section of the cores obtained, the total porosity was 3.3%, indicating that only a small fraction of the concrete volume is occupied by voids. This porosity is distributed between voids due to entrained air (1.9%) and voids due to trapped air (1.4%). The acid-soluble chloride content of the concrete was 0.074% by mass, as determined in accordance with ASTM C1152 [56]. This measured acid-soluble chloride content indicates that the source concrete was not chloride-free and that inherited chloride should be considered when evaluating the RCA for reuse in new concrete [37]. The sulfur trioxide (SO_3_) content in the cement of the pulverized recycled material was 1.15%, which corresponds to an acceptable classification [45].

**Figure 6:**
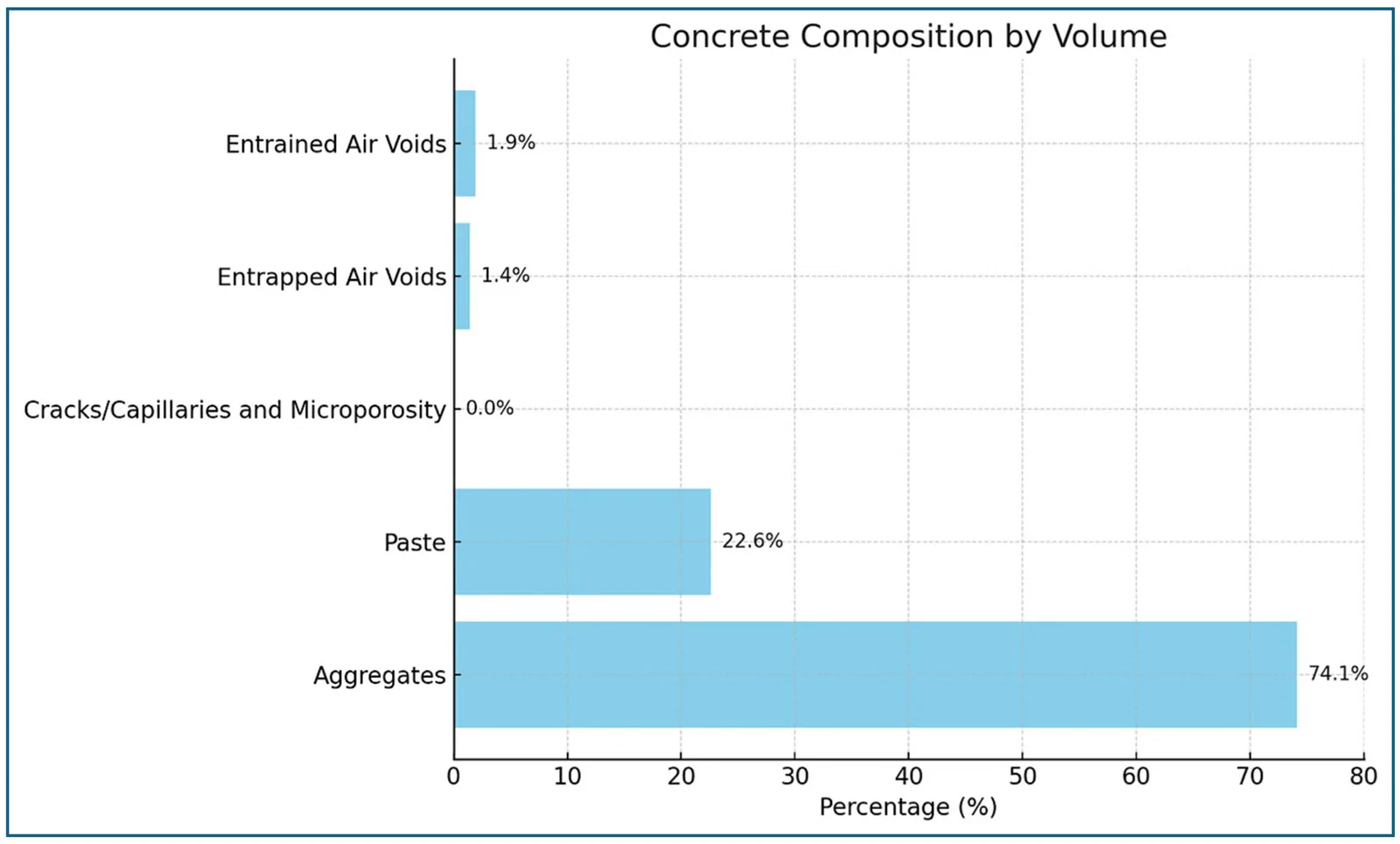
Percentage of primary components in the concrete thin section.

The absorption results for the natural gravel were 4.3%, whereas the crushed construction and CDW coarse aggregate showed an absorption of 6.2%, a higher value than that of the natural aggregate. This behavior is consistent with the wider range of absorption values reported for CDW and RCA in the literature, typically spanning 1.5–2% up to 8–9% and averaging around 5–6% [57,58]. In European Standards, guidance documents recommend adopting water-absorption limits of approximately 3% for coarse aggregates in concrete exposed to demanding environments, primarily as a screening criterion for freeze–thaw resistance rather than as an absolute durability threshold [59,60]. ASTM C127 defines the procedure for determining aggregate absorption but does not, by itself, specify a general maximum acceptance limit for this property [48]. The petrographic study showed that none of the analyzed samples exhibited carbonation fronts within the examined sections. The aggregates varied in size, ranging from fine aggregate (minimum size of 0.1 mm) to coarse aggregate (measured size of 17 mm). Figure 6 shows the percentages of the primary components observed, with the largest proportion (96.7%) corresponding to the sum of aggregates and cement paste.

The types and normalized percentages of aggregates present in the analyzed concrete samples are shown in Table 4. Certain substances in mineral aggregates are chemically or volumetrically unstable and can react with cement alkalis to form expansive alkali–silica gels [61,62]. Of the identified aggregates, 25.9% are considered potentially reactive to ASR, particularly those with high glassy-phase content or micro- to cryptocrystalline textures, such as volcanic glass, quartzite, and chert [62,63]. Within the monomineralic quartz fraction, 2.2% of the grains exhibit undulatory extinction associated with deformation processes, and, following conventional petrographic criteria, these grains are also classified as potentially reactive [62].

**Figure 7:**
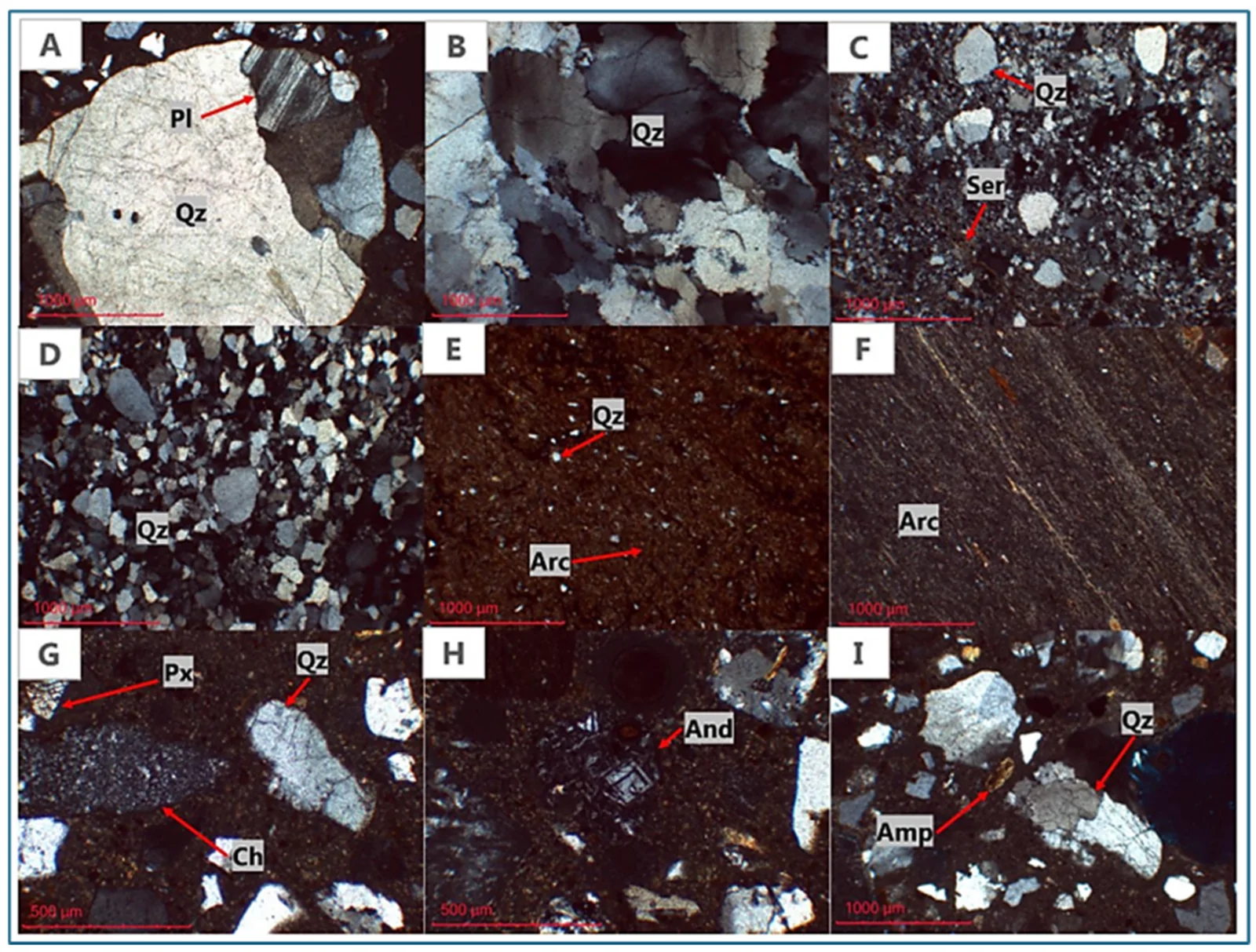
Results of the petrographic analysis of the concrete cores, where (**A**) tonalite composed of quartz crystals (Qz) and plagioclase (Pl) is observed. (**B**) Quartzite composed entirely of quartz crystals (Qz) with sutured edges and undulatory extinction. (**C**) Metarenite composed of deformed and recrystallized quartz crystals (Qz), with sericite crystals (Ser) showing preferential orientation. (**D**) Grain-bearing quartzarenite composed of quartz grains (Qz) with long contacts. (**E**) Reddish ferruginous mud composed of silt-sized quartz (Qz) grains embedded in a clayey matrix (Arc) rich in iron oxides. (**F**) Very finely laminated clay composed of clay minerals (Arc). (**G**) Chert (Ch) composed of cryptocrystalline silica accompanied by quartz (Qz) and pyroxene (Px). (**H**) Hypocrystalline andesite (And) composed of microphenocrysts of plagioclase and amphibole, embedded in a mixed matrix composed of glass and plagioclase microliths. (**I**) Polycrystalline quartz grain (Qz) with undulatory extinction accompanied by amphibole (Amp).

As for the petrographic analysis, Figure 7 shows images of the extracted concrete cores. No reaction edges were evident in the aggregates, and no gel formation was observed.

**Figure 8:**
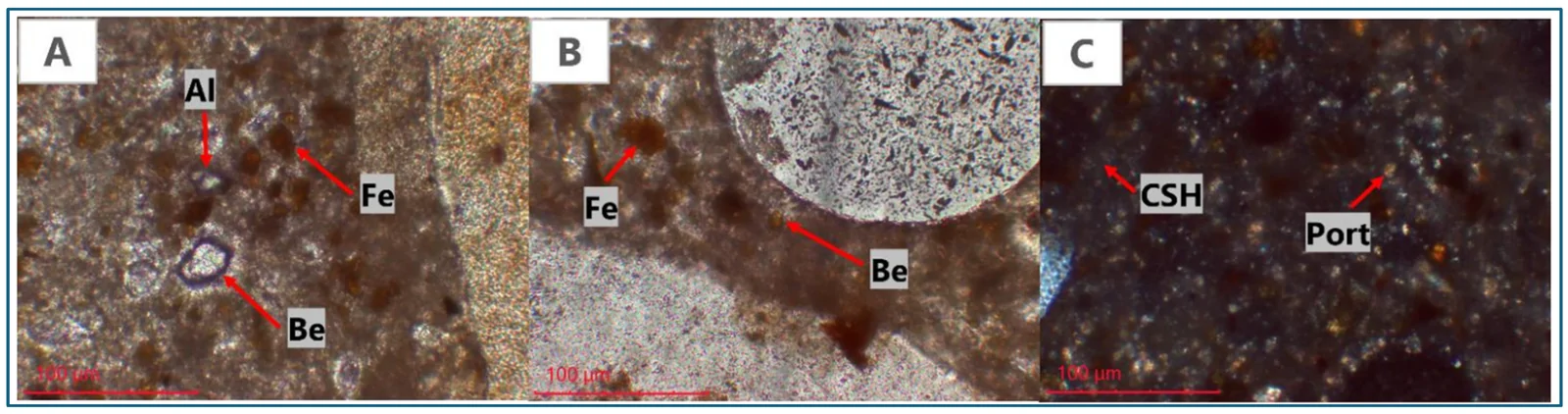
Microstructures formed in the cement paste of the extracted cores. (**A**) Reddish ferrite (Fe) crystals together with rounded, colorless alite (Al) and belite (Be) crystals under plane-polarized light (PPL). (**B**) Pale yellow belite (Be) and reddish ferrite (Fe) crystals under cross-polarized light (XPL). (**C**) Small portlandite (Port) crystals embedded in CSH gel under plane-polarized light (PPL).

The microstructure of the cement paste in the extracted samples shows the presence of ferrite, belite, and portlandite crystals embedded in calcium silicate hydrate (CSH) gel (Figure 8).

**Figure 9:**
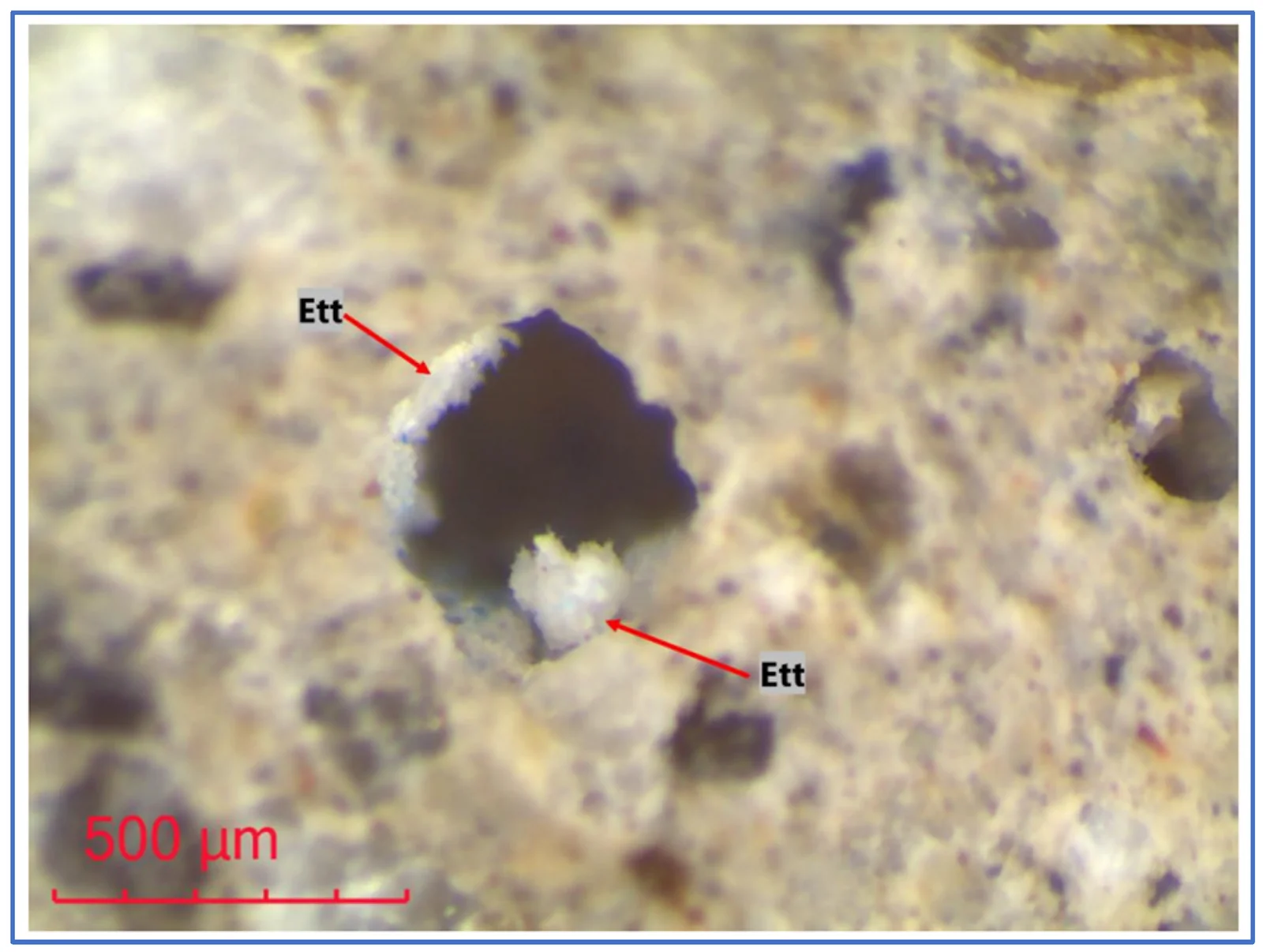
Secondary ettringite (Ett) precipitated in a void, showing its characteristic acicular habit.

Ettringite was observed in a single void approximately 500 μm in diameter (Figure 9). Its occurrence was localized, and no fractures associated with the observed precipitation were identified in the examined section. Consequently, this limited, highly localized ettringite precipitation observed in a single void does not provide evidence of widespread sulfate attack or generalized matrix deterioration, although it highlights the need for cautious interpretation regarding potential localized chemical activity.

The expansion test results for the recycled 3/4″ aggregate classify the material as potentially innocuous, since the expansion values are lower than 0.10% at 16 days, which indicates that the aggregates are not considered potentially deleterious according to the accelerated mortar-bar criteria [64]. Although undulatory extinction was observed in 2.2% of the quartz fraction, and 25.9% of the aggregates were petrographically classified as potentially reactive, recent studies indicate that undulatory extinction alone is not an accurate indicator of ASR susceptibility [62]. Instead, reactivity is more strongly governed by grain-size reduction and the presence of micro- or cryptocrystalline quartz, with quartz crystals approximately 10–60 μm in size generally considered reactive, and those smaller than about 10 μm regarded as highly reactive. In this context, the low expansion values obtained for the RCA are consistent with a limited effective content of equivalent reactive quartz, despite the presence of these potentially reactive features in the microstructure.

### 3.2. Strength Development of RCA-Based Concretes

The compressive strength results for newly produced concrete with a 50% replacement of natural coarse aggregates by RCA and different W/C ratios are given in Table 5. At 3 and 7 days, all mixtures developed strengths below the $f_{c}^{\prime }$ = 21 MPa. At 28 days, however, the mixtures with W/C ratios of 0.61 and 0.47 reached average compressive strengths of 18.00 MPa and 22.77 MPa, respectively, with the latter exceeding the design strength. The results also show a consistent increase in strength with decreasing W/C ratio, confirming the strong influence of mix proportioning on the mechanical performance of concretes with 50% RCA. Despite the expected decrease in strength associated with the use of recycled aggregates, these results show that the mixture with 50% RCA and W/C = 0.47 achieved the specified 28-day $f_{c}^{\prime }$ under the investigated conditions.

## 4. Discussion

### 4.1. Characterization of the Recycled Material

The Colombian regulations and the American Concrete Institute building code indicate that concrete represented by core tests may be considered structurally adequate when the average compressive strength of a group of three cores is at least 85% of the specified compressive strength and no individual core is below 75% of that strength [55,65]. The mean compressive strength of the six tested cores was 23.6 MPa, exceeding 85% of the nominal design strength of 21 MPa. However, individual core results were not uniform: Zone 3 had a corrected compressive strength of 11.8 MPa, below 75% of the nominal design strength (15.75 MPa). Because the cores were obtained from different evaluation zones, including areas with repairs and unknown original design strengths, the results should be interpreted as evidence of heterogeneous in situ concrete quality rather than as a definitive acceptance assessment for the entire pier.

The total porosity determined for the thin section indicates that voids occupy a small fraction of the concrete volume within the examined section. This observation is consistent with a relatively compact air-void system [41]; however, it should not be interpreted as a direct measure of capillary porosity or permeability. This porosity is distributed between entrained air voids (1.9%) and trapped air voids (1.4%). Entrained air voids generally consist of small, relatively well-distributed pores that can improve resistance to freeze–thaw deterioration, whereas trapped air voids are generally larger and more irregular and may result from mixing, placement, or insufficient consolidation [66,67]. The entrained air voids were not uniformly distributed in the examined section. The observed thin-section porosity therefore describes the air-void system in the examined area but does not quantify the capillary pore structure or permeability of the concrete.

The porosity and water absorption of RCA can influence the compressive strength and transport-related properties of concrete incorporating RCA [68]. Based on the results obtained in this study, RCA may be used in concrete mixtures or as base material, provided that mixture proportioning and the intended performance requirements are considered [69].

The acid-soluble chloride content measured according to ASTM C1152 [56] indicates the presence of chloride in the source concrete. Because chloride thresholds and prescriptive limits depend on the chloride fraction, reporting basis, binder composition, exposure condition, and steel–concrete interface [32,37,70], the measured value should not be interpreted as a direct corrosion-initiation threshold or directly compared with limits expressed on a different basis. Although this level does not imply an immediate risk of generalized failure of the demolished pier, it confirms that the recycled material is chloride-contaminated and that any structural reuse must incorporate explicit durability measures in the mix design, cover depth, and, where appropriate, monitoring. However, samples near structural joints contained acid-soluble chloride contents exceeding 0.09% by mass of concrete, indicating non-uniform chloride distribution and a potential concern for localized corrosion exposure [71,72]. The presence of chloride ions (Cl^−^) in the source concrete and in RCA derived from it poses challenges to the structural integrity of reinforced concrete, as they increase the risk of corrosion initiation and may affect chloride penetration and corrosion resistance [37]. While the use of these materials in concrete production is promising from a sustainability perspective [21,30], special attention should be paid to potential impacts on durability [31,37]. Micropores, pre-existing damage, and microcracks in RCA may provide additional chloride-transport paths; however, the magnitude of this effect depends on the RCA characteristics, mix design, exposure conditions, and loading state [73,74].

In practical terms, when chloride-contaminated RCA of this type is considered for use in new reinforced or prestressed concrete, the literature recommends combining several mitigation measures. First, pre-washing or immersion of the RCA in water can reduce leachable chloride; the effectiveness of the treatment should be verified by chloride testing after treatment [37]. Second, mix designs with low W/C ratios and supplementary cementitious materials, such as fly ash or slag, can reduce chloride transport and increase chloride binding under appropriate curing conditions [32,75]. Third, the inherited chlorides from the RCA should be explicitly included in the total chloride budget and assessed against applicable code requirements using a consistent chloride fraction and reporting basis, together with appropriate cover thickness and detailing [25,37]. These measures are particularly relevant when localized chloride enrichment is present near joints or interfaces, where location-specific chloride assessment is required [76].

On the other hand, the sulfur trioxide (SO_3_) content analyzed in the cement present in the pulverized recycled material mixture falls within the acceptable range reported for this type of material [30,37]. Because recycled aggregate concrete may exhibit higher porosity and additional interfacial transition zones, its response to external sulfate exposure depends on the properties of the recycled aggregate, the new cement matrix, and the exposure conditions [77,78]. However, for reuse in marine environments, monitoring is recommended during prolonged exposure to humid or sulfate-rich conditions. Areas with greater humidity exposure showed slightly higher values. It is recommended to apply pre-saturation, hydrophobic agents, or sealing additives to improve the compatibility of RCA with fresh cement [31,32]. In addition, removing residual paste through mechanical attrition can improve the quality of recycled aggregate by reducing porosity and increasing mechanical strength [30].

The higher absorption of the RCA compared with the natural gravel is attributed to its increased porosity and the presence of old mortar adhering to the original aggregate particles, which alters the microstructure and creates a weaker, more permeable interfacial transition zone with the new cement paste [58,79]. As the amount of adhered mortar increases, so does water uptake, which can reduce the workability of fresh mixtures and influence the effective water–cement ratio required to reach a target strength [58,80].

Regarding the petrographic analyses, fine aggregate is predominant and exhibits a uniform distribution. Although the maximum measured size of the coarse aggregates was 20 mm, their average size ranges from 10 mm to 15 mm. The components of the cement paste can be classified into hydrated compounds (such as portlandite and hydrated calcium silicate gel, or CSH gel) and anhydrous compounds, comprising cement elements that have not hydrated, including crystals of alite, belite, ferrite, and clinker. Macroscopically, under plane-polarized light (PPL), the cement paste appears homogeneous, with sporadic anhydrous and portlandite crystals embedded in the CSH gel. Under cross-polarized light (XPL), the CSH gel appears isotropic or pseudo-anisotropic and amorphous, with bright yellow portlandite crystals, gray alite crystals, and rounded belite crystals. These microstructural features indicate that the cement paste retained its fundamental hydration products despite over 20 years of marine exposure. Sedimentary aggregates (55.5%) are present and classified as fragments of mudstones, claystones, quartz arenites, and chert. Metamorphic rocks (13.7%) are also identified and classified as quartzite and metarenite, and igneous rocks (8.4%) are classified as andesite, tonalite, and granite.

Additionally, single-mineral fragments (24.2%) are found, including quartz, plagioclase, amphibole, pyroxene, biotite, iron oxides, and other opaque minerals (see Table 4). The state of preservation of the aggregates indicates that the entire sample shows a low degree of weathering, consistent with fresh rock. However, a small number of aggregates show signs of alteration and/or oxidation, characterized by yellowish-orange tones. Regarding shape, the aggregates exhibit a variety of forms, ranging from subprismatic to discoidal. The coarse particles tend to have sub-angular to sub-rounded edges, while the fine aggregates exhibit contours ranging from very angular to well-rounded. Finally, the expansion tests performed to determine the reactivity of the recycled aggregates indicate that the material meets the criteria for non-reactivity, with expansion percentages below 0.10% at 16 days in most cases [64], indicating innocuous behavior under the accelerated mortar-bar test conditions.

### 4.2. Compressive Strength and Mix Design of New Concretes with RCA

**Figure 10:**
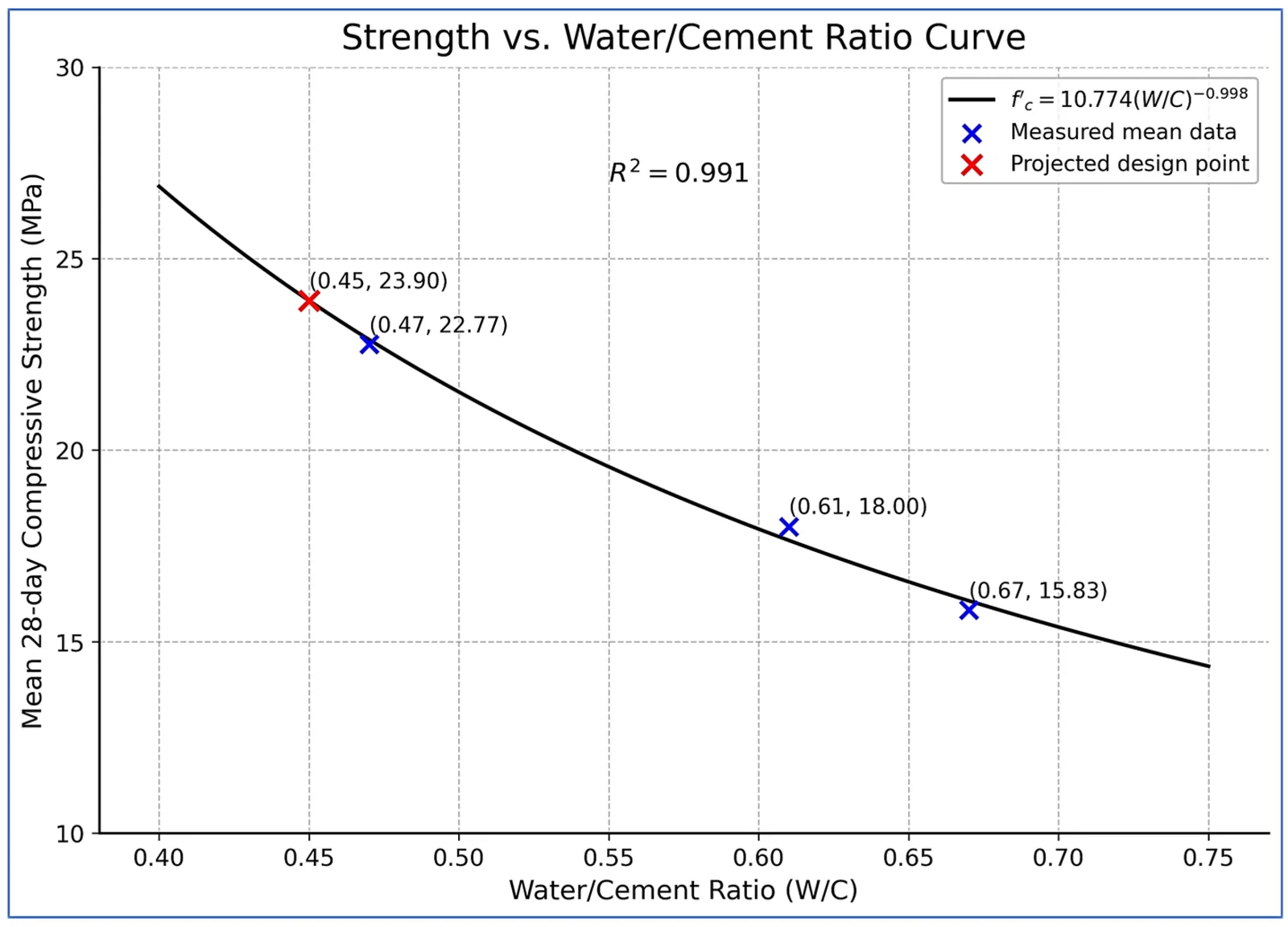
Strength vs. water–cement (W/C) ratio curve for concretes with 50% RCA, fitted to the 28-day compressive strength results of the trial mixes. The red marker denotes a projected point (W/C = 0.45, $f_{c}^{\prime }$ = 23.90 MPa) predicted from the regression and not experimentally validated in this study.

As described in Section 2.3, three trial mixes were prepared with 50% RCA replacement and W/C ratios of 0.67 (M1), 0.61 (M2), and 0.47 (M3). A power-law regression was fitted using the mean 28-day compressive strength obtained from the three replicate specimens for each W/C ratio, as reported in Table 5: 15.83 MPa for M1, 18.00 MPa for M2, and 22.77 MPa for M3. The selected form, $f_{c}^{\prime }=a(W/C{)}^{b}$, is consistent with the inverse relationship between compressive strength and W/C ratio described by Abrams’ law [81]. The model was linearized as $ln(f_{c}^{\prime })=ln(a)+b\cdot ln(W/C)$, and the parameters were determined by ordinary least squares. The resulting empirical relationship was

(1) $$f_{c}^{\prime }=10.774(W/C{)}^{-0.998},$$

with $R^{2}=0.991$ corresponding to the linearized log–log fit (Figure 10). The model estimates that a W/C ratio of approximately 0.512 corresponds to the nominal target strength of 21 MPa. At the statistically projected point of W/C = 0.45, the estimated compressive strength is 23.90 MPa, equivalent to 114% of the nominal design strength. However, this value extrapolates beyond the experimentally evaluated W/C range and requires experimental validation. Because only three W/C levels were investigated, the regression should be regarded as exploratory rather than as a fully validated predictive model.

While a low water–cement (W/C) ratio is essential for creating a denser matrix and higher compressive strength, it is directly associated with more pronounced slump loss, which poses significant challenges for workability [57,82]. Although a reduced W/C ratio can minimize creep and shrinkage in specific applications, such as mechanized molded blocks, an excessively low ratio may leave insufficient water for complete cement hydration, potentially increasing the risk of autogenous shrinkage and cracking [33,83]. Therefore, it is critical to balance workability, often through the use of superplasticizers or compensating water, with rigorous mechanical compaction and controlled curing environments to ensure the structural integrity and long-term durability of the concrete [57,83]. The scientific literature indicates that mixtures with higher RCA content exhibit lower internal cohesion due to RCA’s lower quality relative to natural stone aggregate [30,31,84]. Chloride content and penetration depth may increase if the material is exposed to aggressive marine conditions again [30,32]. To mitigate these risks, future mix designs incorporating RCA from marine-exposed sources should consider treatments such as nanosilica coatings, which react with calcium hydroxide during cement hydration to form additional CSH gel, densifying the matrix, reducing porosity and permeability, and improving resistance to chemical attack and reinforcement corrosion [19,25].

### 4.3. Statistical Analysis of the Correlation Between Compressive Strength and Water–Cement Ratio in Recycled Concretes

The W/C ratio is a key parameter governing concrete compressive strength. Studies have shown that lower W/C values (0.30–0.40) yield denser, lower-porosity concrete with higher compressive strength, whereas higher values (0.50–0.60) tend to reduce strength in both conventional and CDW-based recycled aggregate concretes [57,75,85]. To quantify this trend for the concretes with RCAs studied here, an initial linear regression model was fitted using JASP [54], with compressive strength as the response and curing time and W/C ratio as predictors. Table 6 presents the linear regression results obtained with JASP.

The linear regression analysis showed that compressive strength increases with curing time and decreases with increasing W/C ratio. The fitted equation is

**Figure 11:**
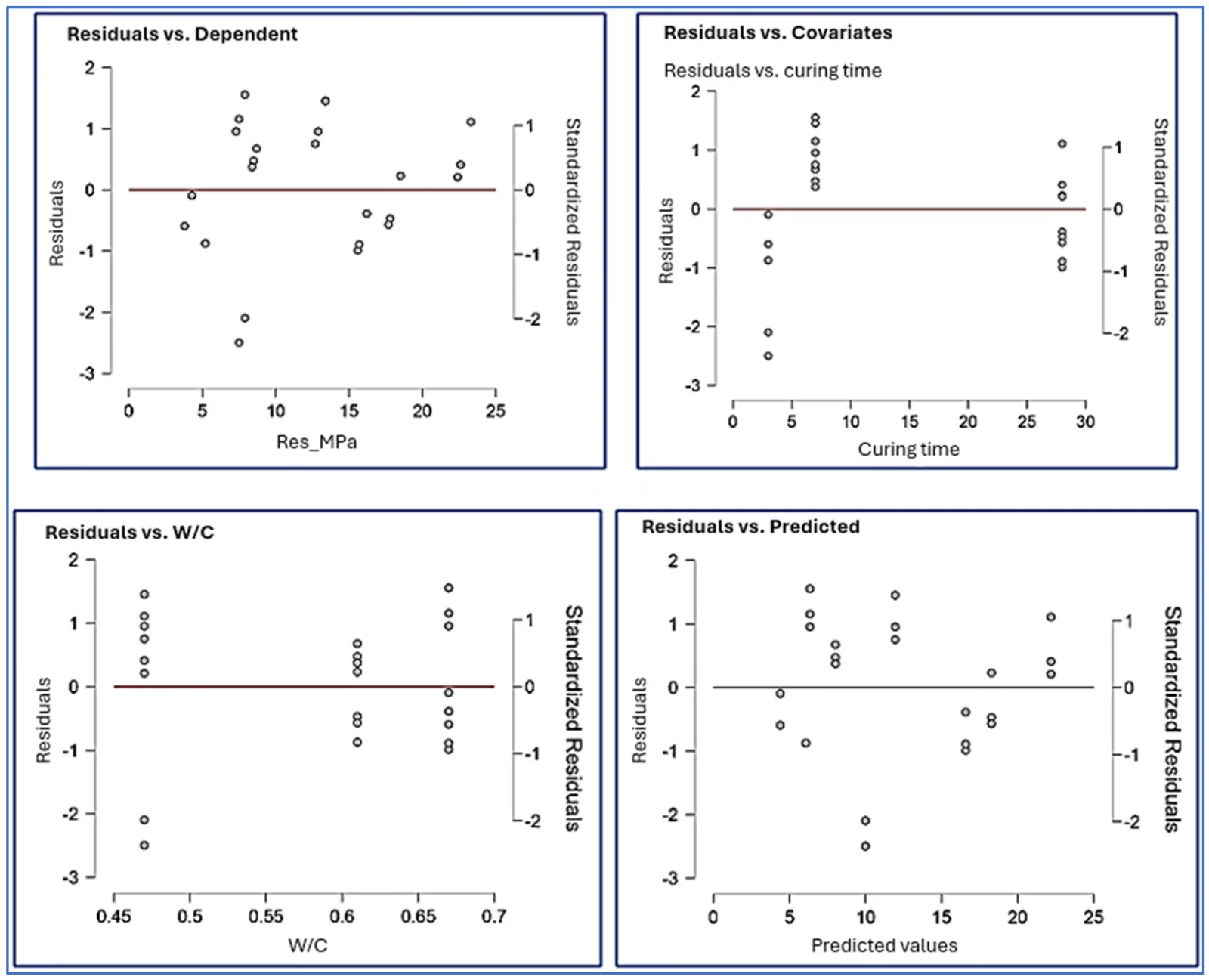
Standardized residual plots for the linear regression model of compressive strength as a function of curing time and water–cement ratio.

Strength (MPa) = 21.694 + 0.488∙X_1_ − 28.006∙X_2_, (2)

where X_1_ = curing time (days) and X_2_ = W/C ratio. The coefficient for Time (0.488 MPa/day) indicates a progressive strength gain with curing, while the coefficient for W/C (−28.006 MPa per unit W/C) reflects a substantial reduction in strength as the water–cement ratio increases. The model explains 97% of the variance in compressive strength (R^2^ = 0.97), with a root mean square error (RMSE) of 1.103 MPa. These model results are consistent with the strength development trends reported in Section 3.2 and confirm that, within the tested range, lower W/C ratios and sufficient curing time are required to meet structural strength levels prescribed by NSR-10 [55] and ACI 318 [65]. The standardized residual plots (Figure 11) show no evident patterns: residuals are randomly scattered around zero for both predictors, indicating that the linearity, homoscedasticity, and independence assumptions of the regression model are reasonably satisfied [86].

Additionally, to capture potential nonlinear effects and interactions between curing time and W/C ratio, a multiple regression model with quadratic and interaction terms was fitted in MINITAB [53]. The multiple regression model fitted with MINITAB is statistically significant (*p* < 0.001) and explains 99.51% of the variance in compressive strength (R^2^ = 99.51%). The fitted equation is

Strength (MPa) = 14.40 + 1.536∙X_1_ − 21.03∙X_2_ − 0.02304∙X_1_^2^ − 0.503∙X_1_∙X_2_, (3)

where X_1_ = curing time (days) and X_2_ = W/C ratio. The inclusion of a quadratic term in X_1_ captures the deceleration of strength gain at later ages. The negative interaction term (−0.503∙X_1_∙X_2_) indicates that the benefit of longer curing is more pronounced at lower W/C values; for instance, at W/C = 0.47, strength increases more rapidly with time than at W/C = 0.67. Because this model is calibrated on only three discrete W/C ratios and 24 cylinders, it should be regarded as a descriptive fit to the observed data within the tested range rather than as a general predictive tool for other W/C values, RCA contents, or exposure conditions.

The inverse relationship between W/C and compressive strength has been widely documented and aligns with Abrams’ water–cement ratio law, which establishes that, for a given age and adequate workability, concrete strength is inversely related to the W/C ratio [81,87], and the nonlinear effect of curing time has been reported for concretes with mechanically treated RCA [88]. Previous studies [19,30,57] indicate that partial replacement of natural coarse aggregate with RCA up to about 50% is technically feasible, maintaining mechanical performance comparable to that of conventional concrete when the mix design, particularly the W/C ratio, is rigorously controlled. In line with this evidence, EN 206:2013 + A2:2021 [89] permits the use of coarse recycled aggregates and, through national provisions based on this standard, typically allows replacement levels up to 50% by mass for selected categories of recycled coarse aggregate in less severe exposure classes, while imposing more restrictive limits (e.g., 20–30%) for lower-quality aggregates or more aggressive environmental classes. These regulatory benchmarks show that, although up to 50% RCA can be compatible with structural performance and durability, allowable substitution rates must be calibrated depending on aggregate quality, exposure class, and the intended structural application. In this setting, the present experimental program focused on a single 50% replacement level, a relatively demanding yet code-consistent substitution range, rather than exploring the full spectrum of RCA contents.

For context, Chen et al. [57] compiled a database of 224 concrete mixes with coarse RCA and 105 reference mixes with natural aggregates, reporting effective W/C ratios between 0.23 and 0.65 (mean 0.44) and compressive strengths between 25.4 and 92.0 MPa (mean 55.0 MPa). The W/C values and strength ranges obtained in this study fall within those reported limits, supporting the representativeness of the experimental dataset. Their study also found that RCA replacement levels up to 20% have a marginal effect on compressive strength, with reductions becoming more gradual beyond that threshold, and that RCA incorporation tends to increase the coefficient of variation in compressive strength. As a cautionary note, Meng et al. [90] advocate that conventional concrete mix design procedures are not directly applicable to mixes with mixed RCAs, as they do not account for the specific characteristics of the recycled fraction; therefore, a purpose-specific design approach is required.

Finally, statistical modeling has been consistently employed as a tool for optimizing the mix of concrete with RCAs [9,57,91]. In this study, the nonlinear model (R^2^ = 99.51%) provided a marginal but physically meaningful improvement over the linear model (R^2^ = 97%), capturing the deceleration of strength gain at later curing ages and the interaction between curing time and W/C ratio, effects that are consistent with the nonlinear nature of strength development in cementitious systems [84,85,90]. Although both models reproduce the observed strength-age-W/C trends very well within the tested range, the literature indicates that nonlinear formulations are generally more robust than purely linear ones when used for interpolation close to the calibration data [57,81,85]. A recognized limitation of both models in this study is the small experimental dataset (*n* = 24 cylinders), which constrains the precision of the coefficient estimates; a larger dataset would improve the reliability and generalizability of the fitted equations. In addition, only three discrete W/C levels were investigated, so the nonlinear model must be regarded as a descriptive fit to these specific mixes and ages rather than a predictive tool for extrapolation to other W/C ratios or replacement levels.

Future studies should explore nonlinear regression and data-driven approaches, such as artificial neural networks and machine learning algorithms, to predict the compressive strength of RCA-based concrete from marine-exposed structures across a wider range of W/C ratios, RCA replacement levels, and curing ages. Their effectiveness depends on larger datasets, as shown by Deshpande et al. [92], who found that, for concrete with RCA strength modeling, data-driven methods outperform linear regression, but their generalizability depends on the size and diversity of the training data.

## 5. Conclusions

The results indicate that, with proper mix proportioning and control of the water–cement ratio, concrete samples with 50% recycled concrete aggregates (RCAs) derived from a pier exposed for over 20 years to a tropical marine environment can achieve 28-day compressive strengths close to or exceeding the 21 MPa design value established in the Colombian NSR-10, particularly for W/C ≤ 0.47. This performance supports their use in structural elements with moderate demand, provided that mix design and quality control are adapted to the properties of the recycled aggregate.

Recycled aggregates absorb more water than natural ones, affecting concrete workability and increasing water demand. Pre-treatments like pre-saturation or admixtures can improve workability and reduce variability in recycled concrete properties.

Compressive strength analysis of concrete with 50% RCA and W/C ratios of 0.67, 0.61, and 0.47 showed a systematic increase in strength with decreasing W/C, confirming that lower W/C values within the tested range are necessary to reach or exceed the nominal design strength. A power-law regression between W/C and 28-day compressive strength identified a projected low-W/C region around 0.45, providing an exploratory baseline for future optimization of mixes with 50% RCA.

Regarding durability, the RCAs from the demolished pier showed low total porosity, suggesting a dense structure that enhances resistance to chloride penetration. The measured acid-soluble chloride content indicates that the source concrete and the derived RCA are chloride-contaminated and cannot be treated as chloride-free materials, so their structural reuse requires explicit durability design and, where necessary, monitoring strategies. Additionally, localized accumulation near joints may increase the vulnerability of specific zones. Sulfate analysis determined a SO_3_ content that is classified as acceptable. However, periodic monitoring is recommended in prolonged exposures to humid environments and high sulfate concentrations. Furthermore, alkali–silica reactivity tests revealed expansions below the threshold values used to classify aggregates as potentially deleterious, classifying the recycled aggregates as non-reactive and ensuring their long-term chemical stability.

Implementing a circular economy for CDW management, like the SBP, lowers demand for natural aggregates and environmental impacts from quarrying. It valorizes RCA from marine structures as a raw material. The case study shows that end-of-life concrete in marine environments can be reused, supporting SDGs 9 and 12 by fostering sustainable infrastructure and circular material use, and SDG 13 by reducing embodied emissions from quarrying, processing, and disposal, aiding climate mitigation.

Future studies should apply a structured design-of-experiments approach (such as a full factorial or response surface design) to simultaneously optimize W/C ratios, RCA replacement levels, and curing time. This approach was not pursued in the present study due to resource constraints, but it would enable the development of predictive models for the compressive strength of concretes incorporating RCAs from marine-exposed structures. Other potentially relevant variables, such as the use of supplementary cementitious materials, RCA pre-treatment strategies, and exposure conditions representative of specific marine exposure classes, were not assessed in this study and should be incorporated into future experimental programs. In addition, the present results are limited to RCA obtained from a single offshore structure, one cement type, a single coarse aggregate replacement level (50%), and one tropical marine exposure condition, so their applicability to other source concretes, binders, replacement ratios, and environments should be evaluated with broader datasets. Consequently, the results at 50% RCA replacement cannot be directly extrapolated to other replacement levels (e.g., 30%, 70%, or 100%), which may exhibit different strength–durability relationships and require dedicated experimental programs for characterization.

## Figures and Tables

**Table 1:** Characteristics of the aggregates employed in the concrete fabrication.

Physical Characteristics of Aggregates	Quarry Fine Sand	Quarry Gravel 3/4″	50:50 RCA Mix 3/8″ and 1/2″
Fineness module	2.35	-	-
Maximum size (mm)	-	19	25
Maximum nominal size (mm)	-	12.5	19
Organic matter content	1	-	-
Absorption (%)	0.6	4.3	6.2
Relative Density (Oven-Dry—OD)	2.58	2.27	2.15
Relative Density (Saturated-Surface-Dry—SSD)	2.6	2.37	2.29
Relative Density (Apparent)	2.62	2.52	2.49
SSD Loose Volumetric Density (kg/m^3^)	1450	1320	1280
Buffered Volume Density SSD (kg/m^3^)	1610	1460	1390
Loose aggregate voids (%)	45	50	51
Tamped aggregate voids (%)	39	44	47
Sand equivalent	73	-	-

**Table 2:** Mix proportions and aggregate properties for the concrete mixture.

Material	Content (kg/m^3^)	Density (kg/L)	Absorption (%)	Porosity (%)	Loose Bulk Density (kg/m^3^)	Compacted Bulk Density (kg/m^3^)	Voids in Loose Aggregate (%)	Voids in Compacted Aggregate (%)
Argos Type I cement	444	-	-	-	-	-	-	-
Water	200 *	-	-	-	-	-	-	-
Sand	629	2.62	0.6	-	1450	1610	45	39
Gravel 3/4″ (50%)	480	2.52	4.3	-	1320	1460	50	44
RCA 3/4″ (50%)	480	2.49	6.2	3.30	1280	1390	51	47

* Water content expressed in liters per cubic meter, assuming a density of 1.0 kg/L. Only the “Content (kg/m^3^)” column corresponds to mixture proportions; all other columns report aggregate properties used to support the mix-design process.

**Table 3:** Geometric characteristics, maximum load, and corrected compressive strength of concrete cores extracted in situ.

Location	Length	Diameter	L/D	L/D Correction Factor	Maximum Load	Compressive Strength	
	(mm)	(mm)			(kN)	(MPa)	(kg/cm^2^)
Zone 1	125.2	63.2	1.98	1.00	106.2	33.9	345
Zone 2	128.4	63.0	2.04	1.00	92.7	29.7	303
Zone 3	127.9	63.1	2.03	1.00	37.0	11.8	121
Zone 4	128.2	62.8	2.04	1.00	93.4	30.1	307
Zone 5a	127.4	63.0	2.02	1.00	53.1	17.0	173
Zone 5b	67.0	63.1	1.06	0.89	66.3	18.8	192

**Table 4:** Types of aggregates and percentages observed by petrographic analysis.

Origin	Aggregates	Percentage (%)
Igneous	Tonalite	2.10
	Granite	0.50
	Volcanic glass	2.10
	Andesite	1.90
Sedimentary	Lodolite	20.90
	Clay	9.10
	Sandstone Quartz	17.30
	Chert	8.20
Metamotphic	Quartzite	8.20
	Metarenite	5.50
Monominerals		24.20
Total		100.00

**Table 5:** Data from the results of the compressive strength tests of Trial Mix Cylinders. Design compressive strength for all mixes: $f_{c}^{\prime }$ = 21 MPa.

Case (Mix/Cylinder)	Age (Days)	Maximum Load (kN)	Compressive Strength			Water/Cement Ratio (W/C)
			MPa	(kg/cm^2^)	%	
M1C1	3	31.04	3.80	38.70	18.10	0.67
M1C2	3	34.90	4.30	43.60	20.30	0.67
M1C3	7	61.22	7.50	76.40	35.70	0.67
M1C4	7	64.80	7.90	80.10	37.40	0.67
M1C5	7	59.86	7.30	74.70	34.90	0.67
M1C6	28	127.80	15.60	158.80	74.20	0.67
M1C7	28	132.66	16.20	165.50	77.30	0.67
M1C8	28	128.66	15.70	160.60	75.00	0.67
M2C1	3	42.68	5.20	53.30	24.90	0.61
M2C2	3	42.88	5.20	53.50	25.00	0.61
M2C3	7	70.82	8.70	88.40	41.30	0.61
M2C4	7	69.15	8.50	86.30	40.30	0.61
M2C5	7	68.31	8.40	85.20	39.80	0.61
M2C6	28	145.79	17.80	181.89	85.00	0.61
M2C7	28	151.54	18.50	189.10	88.30	0.61
M2C8	28	144.37	17.70	180.20	84.10	0.61
M3C1	3	64.9	7.90	81.00	37.80	0.47
M3C2	3	61.04	7.50	76.20	35.60	0.47
M3C3	7	105.61	12.90	131.80	61.50	0.47
M3C4	7	104.18	12.70	130.00	60.70	0.47
M3C5	7	109.86	13.40	137.10	64.00	0.47
M3C6	28	182.66	22.40	227.90	106.40	0.47
M3C7	28	190.28	23.30	237.50	110.90	0.47
M3C8	28	185.03	22.60	230.90	107.80	0.47

**Table 6:** Linear regression analysis with JASP.

Row Type	Model	Source/Predictor	R^2^	df	F	Coefficient	Std. Error	t	*p*-Value
Model	H_0_	Intercept-only	0.00						
Model	H_1_	W/C, Age (days)	0.97						
ANOVA	H_1_	Regression		2	341				<0.001
ANOVA	H_1_	Residual		21					
ANOVA	H_1_	Total		23					
Coefficient	H_0_	Intercept				12.125	1.245	9.742	<0.001
Coefficient	H_1_	Intercept				21.694	1.608	13.490	<0.001
Coefficient	H_1_	W/C				−28.006	2.686	−10.425	<0.001
Coefficient	H_1_	Age (days)				0.488	0.020	23.945	<0.001

Note: The response variable is compressive strength (MPa). H_0_ is the intercept-only model; H_1_ includes W/C ratio and curing age (days) as predictors. The 95% confidence intervals for the H_1_ coefficients, computed in JASP, are as follows: Intercept: [18.349, 25.038]; W/C: [−33.593, −22.420]; Age (days): [0.445, 0.530].

## Data Availability

The original contributions presented in this study are included in the article. Further inquiries can be directed to the corresponding authors.

## Abbreviations

The following abbreviations are used in this manuscript:

$f_{c}^{\prime }$	Design compressive strength of concrete
ASR	Alkali–silica reaction
ASTM	American Society for Testing and Materials
CDW	Construction and demolition waste
CSH	Calcium silicate hydrate
NSR-10	Colombian Building Code for Seismic Resistant Design
RCA	Recycled concrete aggregate
SBP	Shore Base Pier
W/C	Water–cement ratio
